# Microgeographic Heterogeneity of Border Malaria During Elimination Phase, Yunnan Province, China, 2011–2013

**DOI:** 10.3201/eid2208.150390

**Published:** 2016-08

**Authors:** Xin Xu, Guofa Zhou, Ying Wang, Yue Hu, Yonghua Ruan, Qi Fan, Zhaoqing Yang, Guiyun Yan, Liwang Cui

**Affiliations:** Kunming Medical University, Kunming, China (X. Xu, Y. Hu, Y. Ruan, Z. Yang);; University of California, Irvine, California, USA (G. Zhou, G. Yan);; Institute of Tropical Medicine, Third Military Medical University, Chongqing, China (Y. Wang);; Dalian Institute of Biotechnology, Dalian, China (Q. Fan);; Pennsylvania State University, University Park, Pennsylvania, USA (L. Cui)

**Keywords:** Plasmodium falciparum, Plasmodium vivax, malaria, fine-scale, spatial heterogeneity, spatial clustering, international border, China, Myanmar, parasites, mosquitoes, vector-borne infections

## Abstract

To identify township-level high-risk foci of malaria transmission in Yunnan Province, China, along the international border, we retrospectively reviewed data collected in hospitals and clinics of 58 townships in 4 counties during 2011–2013. We analyzed spatiotemporal distribution, especially hot spots of confirmed malaria, using geographic information systems and Getis-Ord G_i_*(d) cluster analysis. Malaria incidence, transmission seasonality, and *Plasmodium vivax*:*P. falciparum* ratio remained almost unchanged from 2011 to 2013, but heterogeneity in distribution increased. The number of townships with confirmed malaria decreased significantly during the 3 years; incidence became increasingly concentrated within a few townships. High-/low-incidence clusters of *P. falciparum* shifted in location and size every year, whereas the locations of high-incidence *P. vivax* townships remained unchanged. All high-incidence clusters were located along the China–Myanmar border. Because of increasing heterogeneity in malaria distribution, microgeographic analysis of malaria transmission hot spots provided useful information for designing targeted malaria intervention during the elimination phase.

## Abstract 2

Malaria was concentrated in a few townships along the China–Myanmar border.

Malaria, one of the most devastating infectious diseases, creates an enormous public health burden in the developing world (*1*). Since 2000, increased financial support has strengthened malaria control programs, leading to substantially reduced malaria incidence and death rates, even in the high-transmission areas of sub-Saharan Africa (*1*). The estimated worldwide malaria death rate declined by 45% during 2000–2012. Of the 97 countries with malaria transmission in 2013, twelve are in the preelimination phase and 7, including China, are in the elimination phase. In early 2009, China’s Ministry of Health presented its Revised National Malaria Strategy 2010–2015; this strategy was followed by the Malaria Elimination Action Plan for 2010–2020, in which the Ministry of Health laid out a strategy to eliminate malaria by 2020 (*2*,*3*).

Control efforts, guided by the 1–3-7 strategy (reporting a malaria case within 1 day; confirming, treating, and investigating the case within 3 days; and delivering an appropriate public health response to prevent further transmission within 7 days) have drastically reduced malaria incidence in central China (*4*). As a result, malaria transmission is restricted to the southwestern Yunnan Province along the international borders (*5*–*8*). Currently, *Plasmodium vivax* is the predominant species of malaria parasites in China, and autochthonous *P. falciparum* occurs only in Yunnan Province (*7*,*8*). 

In 2012, Yunnan Province reported an annual malaria incidence of 7.4 cases/100,000 population (*8*). Yunnan Province borders 3 malaria-endemic countries: Myanmar, Laos, and Vietnam. Previous studies found that cross-border migration from Myanmar was the major source of importation/reintroduction of *P. falciparum* malaria in Yunnan Province (*9*). Therefore, the control strategy during the elimination phase must focus on eliminating local transmission and cross-border introduction.

Elimination strategies can differ profoundly from control strategies because they require prospective, accurate identification of transmission foci and rapid control responses (*10*–*13*). Earlier studies in China relied exclusively on retrospective data acquired from county-level hospital records (*5*–*8*). The retrospective nature of these studies raises questions about diagnostic accuracy, whereas the county-level epidemiologic data provide limited spatial resolution. A county in China typically comprises many townships, sometimes ≈100 villages and totaling ≈1 million persons, and it might span ≈100 km (*14*–*17*), which limit the usefulness of county-level risk assessment for guiding targeted malaria control and local malaria elimination. Thus, for spatially heterogeneous malaria transmission, finer-scale mapping is essential for deploying elimination measures.

We aimed to use prospectively confirmed malaria data to identify high-risk foci of malaria transmission at the township level in Yunnan Province along the international border. Specifically, we wanted to locate the transmission hot spots and determine whether malaria transmission is heterogeneous at the township level. Our goal was to provide data to help guide targeted malaria control response during the malaria elimination phase.

## Methods

### Study Area

**Figure 1:**
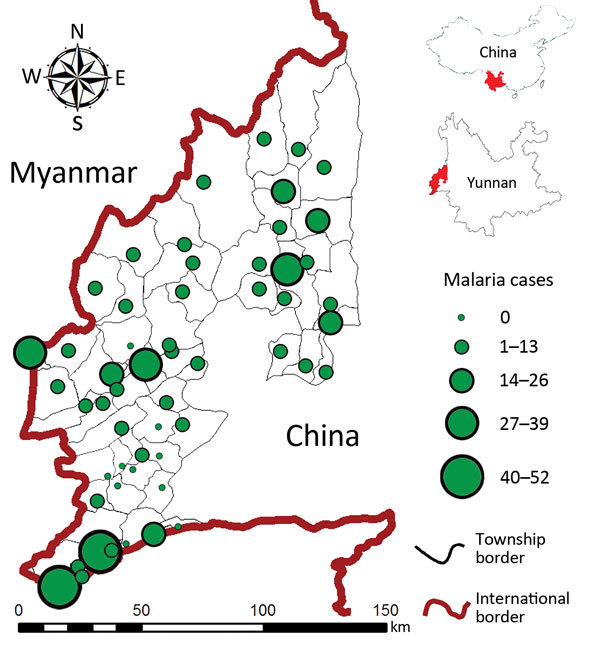
Locations of hospitals and healthcare centers (center of each circle) and total confirmed malaria cases in each township, Yunnan Province, China, 2011–2013.

The study area comprised 58 townships in 4 counties (Tengchong, Yingjiang, Longchuan, and Ruili) along the China–Myanmar border in Yunnan Province (Figure 1). Each township has 1 government-run healthcare center consisting of an inpatient hospital and other administrative facilities and sometimes a few small village-level clinics. In accordance with government policy, malaria diagnosis and treatment are free, regardless of the patient’s nationality or origin of residence. The study area spans 13,200 km^2^; the population was ≈1.3 million in 2010. Approximately 85% of residents live in rural areas, and most are farmers. The climate is subtropical; average maximum/minimum temperatures ranges from 13/4°C in January to 34/24°C in July. Annual rainfall in the study area is ≈2,200 mm, with 1 rainy season during April–July but no clear dry season. The topography is characterized by mountains separated by small, long, narrow basins dominated by rice fields. Among the 4 counties, Ruili is located in the largest basin of the study area. In 2012, Tengchong, Yingjiang, and Ruili counties were among China’s leading 5 counties in malaria incidence (*8*).

### Data Collection

We applied a prospective surveillance method and included all patients with fever who sought care at 58 local health centers and 4 hospitals in the study area during January 2011–December 2013. Local doctors screened persons with fever at local hospitals/clinics for malaria symptoms after patients signed consent or assent (for minors <18 years of age) forms (i.e., screening for suspected cases). A suspected malaria case was defined as malaria-related symptoms (fever with axillary temperature >37.5°C, chills, severe malaise, headache, or vomiting) at examination or 1–2 days before examination. In brief, before sample collection, study procedures (i.e., sampling procedures, study benefits, and potential risks and discomforts) were explained to patients. Before samples were collected, demographic data such as sex, age, home address and name of participant, travel history during the preceding 14 days, malaria infections during the preceding 6 months and prescriptions obtained, and use of preventive measures were recorded. Identification numbers were used in the final analysis, and each patient’s real identity and address was kept confidential. Blood samples were collected by finger-pricking. Labeled slides with thin and thick blood smears were prepared for microscopic *Plasmodium* species identification and parasite density counts. Thick and thin blood films were stained with 10% Giemsa and examined according to a previously described method (*18*). The hospitals where the patients were admitted confirmed all reported cases. The blood slides were reexamined by 3 experienced microscopists, who provided the final confirmation of malaria cases. For slides that were confirmed positive, the asexual parasite density and gametocyte density were scored against 200 leukocytes. Confirmed malaria was defined as malaria-related symptoms at examination or 1–2 days before examination and a *Plasmodium*-positive blood smear confirmed by microscopy. Persons in whom malaria was diagnosed were treated in accordance with national malaria treatment guidelines (http://www.who.int/malaria/publications/atoz/9789241549127/en/).

### Data Analysis

We calculated the malaria incidence rate as cases per 100,000 population per year or month based on 2010 population census data. Nonlocal residents (e.g., visitors and travelers) were excluded from incidence rate calculation. We examined year-to-year differences in the number of townships where confirmed malaria was absent using a χ^2^ test. We tested township-level year-to-year differences in incidence using the Tukey-Kramer honest significant difference (HSD) of analysis of variance (ANOVA) post hoc test. *P. vivax* and *P. falciparum* case ratio for each year was also calculated.

We mapped spatial distribution of incidence rate at the township level using ArcGIS 10 (ESRI, Redlands, CA, USA). Spatial heterogeneity of confirmed malaria cases was measured with median incidence rate, range of incidence rate, Kurtosis, skewness, and coefficient of variation of incidence among townships. To determine whether the distance from the hospital or healthcare centers to the nearest border affected malaria incidence rate, we calculated distances from the hospital or healthcare centers to the nearest border using ArcGIS and divided them into 4 categories (0–9, 10–29, 30–49, and >50 km); average incidence rate of *P. falciparum* and *P. vivax* were calculated for each distance group; and differences in incidence rate among different distance groups were compared using the Tukey-Kramer HSD of ANOVA post hoc test. To identify spatial clusters that might characterize the distribution of malaria at the China–Myanmar border, we used the Getis-Ord local G_i_*(d) test to determine the sizes and locations of high- and low-incidence clusters at the township level (*19*,*20*). Getis-Ord G_i_*(d) statistics have been commonly used for disease transmission hot spot analysis (*14*,*21*–*24*). We examined hot spots separately for *P. falciparum* and *P. vivax* and annually so we could track temporal changes in transmission hot spot.

### Ethical Statement

The institutional review boards of Kunming Medical University (Kunming, China); University of California, Irvine (Irvine, CA, USA); and Pennsylvania State University (University Park, PA, USA) approved the study. We obtained written informed consent or assent (for minors <18 years of age) from all persons or parents or guardians who were willing to participate in the study.

## Results

### Descriptive Statistics of Confirmed Malaria

Blood smear examination by microscopy confirmed 468 malaria cases from the 915 suspected cases screened from ≈23,000 febrile persons. The annual confirmed malaria incidence rate was 13.1 cases/100,000 population. We detected all 4 human *Plasmodium* species; *P. vivax* and *P. falciparum* accounted for 334 (71.4%) and 123 (26.3%), respectively. We found only 1 (0.2%) *P. malariae* and 4 (0.9%) *P*. *ovale* infections. Six patients carried mixed infections by *P. falciparum* and *P. vivax*.

### Temporal Trend in Confirmed Malaria

**Figure 2:**
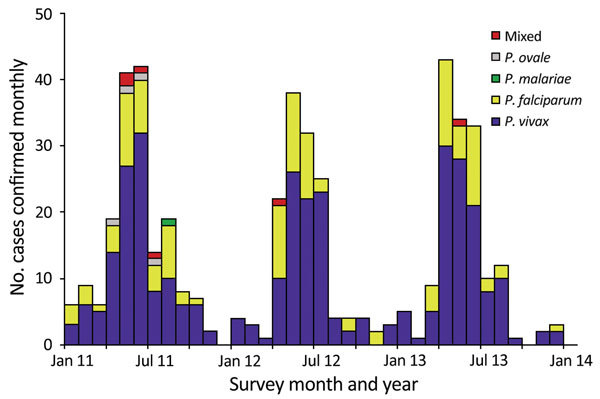
Monthly number of confirmed malaria cases of different *Plasmodium* species, Yunnan Province, China, 2011–2013.

**Figure 3:**
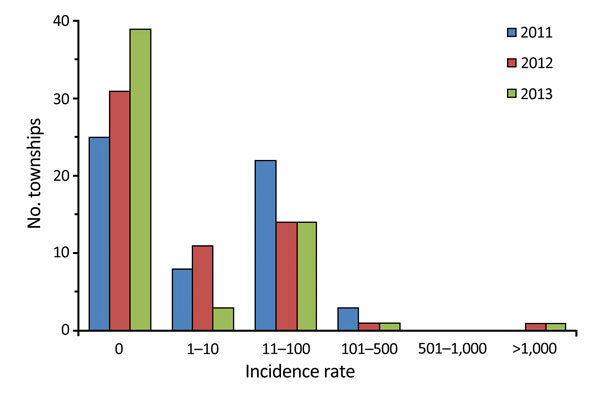
Malaria incidence (cases per 100,000 population), by year, Yunnan Province, China, 2011–2013.

Confirmed malaria incidence showed strong seasonality and peaked during April–August each year (Figure 2). In December 2011 and October 2013, confirmed malaria infections were not detected in the study area, whereas during the peak months, incidence increased sharply to ≈40 cases/month (3.3 cases/100,000 population/month) in each year of the study. *P. vivax* and *P. falciparum* parasites were detected nearly every month, but the other parasites and mixed infections were detected only during the high-transmission season (Figure 2). The *P. vivax* and *P. falciparum* case ratio remained almost unchanged: 2.5 for 2011, 2.6 for 2012, and 2.8 for 2013. The percentage of townships with no confirmed malaria increased from 43.1% in 2011 to 53.4% in 2012 and 67.2% in 2013 (Figure 3; Table), indicating a continuous reduction in malaria transmission in the area. This reduction also is reflected in the median incidence rates; the 0 median incidence rate in 2012 and 2013 indicated that ≈50% of townships had no confirmed malaria. The increased mean incidence rates from 2011 to 2013 reflected the increased heterogeneity of the distribution.

**Table:** Measurement of heterogeneous distribution of confirmed township-level malaria incidence rate, Yunnan Province, China, January 2011–December 2013

Parameter	Year of surveillance		
	2011	2012	2013
No. townships with confirmed malaria*	33	27	19
No. malaria cases	170	144	149
Incidence rate†			
Mean (95% CI)	17.9 (8.2–27.6)	29.1 (0–68.3)	31.7 (0–71.3)
Median	5.4	0	0
Range	200	1133.3	1133.3
Kurtosis	14.3	55.2	52.6
Skewness	3.6	7.4	7.1
Coefficient of variation	205.6	513.9	475.3

*Significant differences (p<0.05) were determined by χ^2^ test. The differences between 2011 and 2012 and between 2012 and 2013 were not significant; however, the difference between 2011 and 2013 was significant. †Cases/100,000 population/year.

### Spatial Heterogeneity in Confirmed Malaria

Malaria was increasingly concentrated in fewer townships, and the heterogeneous level increased among townships over time (Table). The annual township-level incidence rate of malaria ranged from 0 to 200.0 cases/100,000 population in 2011 and from 0 to 1,133.3 cases/100,000 population in 2012 and 2013; however, the average incidence rate changed only from 17.9 to 31.7 cases/100,000 population annually from 2011 to 2013 (Table), and the increased mean incidence rate was caused primarily by a few extremely high-incidence townships. We detected malaria only from 33 townships during 2011, 27 during 2012, and 19 during 2013 (Figure 3). Coefficients of variation were well above 100% in all 3 years (Table). Spatial heterogeneity in incidence rate increased significantly over time (Table). These findings indicate a significantly heterogeneous distribution in township-level malaria incidence. Post hoc comparison indicated that differences in the annual average incidence rate at the township level were statistically insignificant among the 3 years (Tukey-Kramer HSD p>0.05), which indicated that the increasing mean annual incidences among townships from 2011 to 2013 most likely resulted from extreme variation in incidence rate among townships.

### Confirmed Malaria versus Distance to the Border

**Figure 4:**
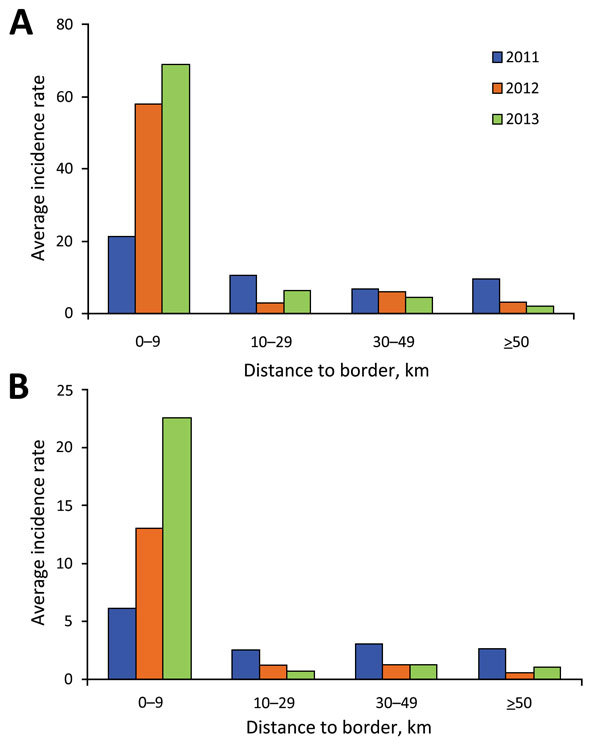
Malaria incidence (cases per 100,000 population) and distance to the nearest border, by year, Yunnan Province, China, 2011–2013. A) *Plasmodium vivax*. B) *P. falciparum*.

The distance versus incidence rate analysis indicated that malaria incidence was 2- to 30-fold higher in areas within 10 km of the international border than it was in areas farther from the border, and the difference in incidence rate between the 2 categories increased during the 3 years. Within 10 km of the border, the annual *P. vivax* incidence was 21.48 during 2011, 57.89 during 2012, and 68.77 during 2013; for *P. falciparum*, incidence was 6.11during 2011, 13.04 during 2012, and 22.62 during 2013 (Figure 4). This finding indicated that confirmed malaria incidence was increasingly aggregated along the border. However, because of the huge variances in each group, statistical tests did not show any significant differences in the incidence rate among different distance groups (data not shown).

### Hot Spots of Confirmed Malaria

**Figure 5:**
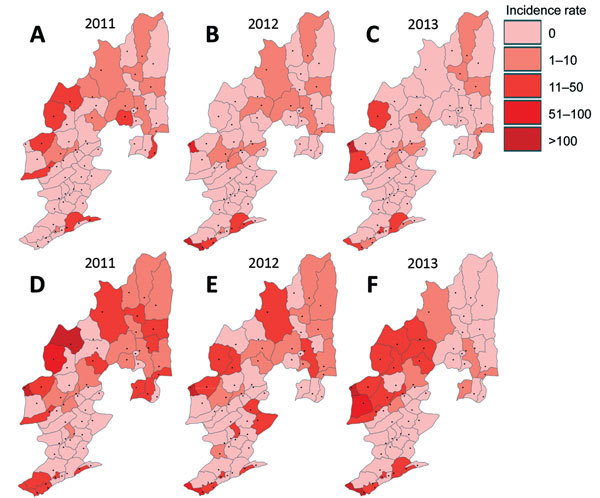
Distribution of township-level malaria incidence rate (cases per 100,000 population), Yunnan Province, China, 2011–2013. A–C) *Plasmodium falciparum*. D–F) *P. vivax*.

**Figure 6:**
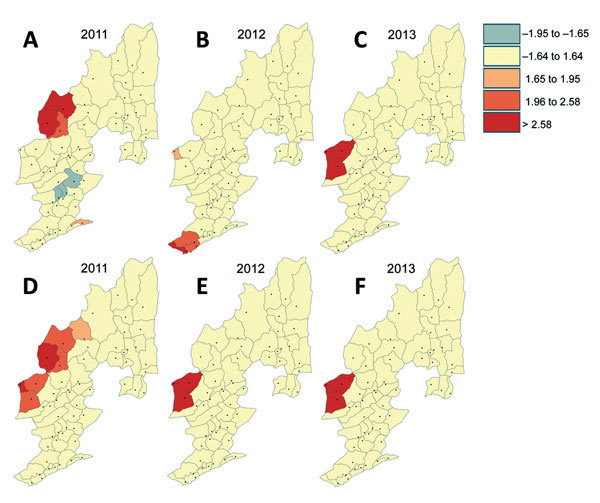
Clusters of low and high malaria incidence rates (cases per 100,000 population) detected at the township level and their shift over time, Yunnan Province, China, 2011–2013. A–C) *Plasmodium falciparum*. D–F) *P. vivax*.

The distribution of malaria incidence rates (Figure 5) and the clusters of low and high incidence rates (Figure 6) showing hot spots of confirmed malaria varied throughout the years at the township level. Nevertheless, the transmission hot spots were generally located close to the international border.

The distribution maps of malaria incidence rates showed large spatial variation at the township level (Table; Figure 5). Townships with high incidences of *P. vivax* and *P. falciparum* were located mostly along the international border. However, many townships along the border had no confirmed malaria (Figure 5), illustrating the complex heterogeneous nature of malaria transmission in the border area. Compared with 2011, the geographic range of malaria incidence had shrunk in 2013. Clinical *P. falciparum* malaria cases were extremely scarce in 2013 (Figure 5, panel C), and *P. vivax* malaria cases were more aggregated in the central area along the border (Figure 5, panel F).

Results of the Getis-Ord G_i_*(d) test indicated that all high-incidence clusters were located along the China–Myanmar border, namely Nabang, Xima, and Tongbiguan townships (Figure 6). The population of these 3 townships is ≈46,400, and the area is ≈1,530 km^2^. However, for *P. falciparum* malaria, both the sizes and the exact locations of high- and low-incidence clusters shifted over time (Figure 6, panel A). On the other hand, high-incidence clusters of *P. vivax* remained in the same areas, although the size of clusters shrank from 2011 to 2012–2013. Coincidentally, high-incidence clusters comprised exactly the same townships for both *P. falciparum* and *P. vivax* in 2013 (Figure 6, panels C, F).

## Discussion

China successfully reduced illness and death from malaria during the 1980s–2000s and is now in the malaria elimination phase. Strategies to achieve and maintain malaria elimination should concentrate on identifying and eliminating transmission foci through passive and active methods of case detection (*12*). As malaria transmission declines, the number and sizes of the infection foci are shrinking. Thus, finer-scale transmission maps are required to identify small transmission hot spots. Because the township is the smallest administrative unit in China that has government-run healthcare centers, transmission mapping at the township level might be the best approach for elimination planning.

In Yunnan Province, *P. vivax*, which is difficult to eliminate, has become the predominant parasite species. Therefore, as overall malaria incidence rates fell in a region, we would expect the proportion of *P. vivax* cases to increase (*25*). Previous studies found that clinical *P. falciparum* malaria in Yunnan Province accounted for ≈15% of total cases during 1991–2006 and 22% during 2001–2005 (*26*,*27*). However, we did not find further decrease in the proportion of *P. falciparum* malaria during 2011–2013, which accounted for ≈27% of total clinical cases. Most *P. falciparum* cases occurred along the international border. Importation probably explains this unusual increase in the *P. falciparum* proportion. A previous study conducted in the same area found that clinical *P. falciparum* malaria was significantly associated with cross-border travel, especially travel to Myanmar (*9*).

Because malaria transmission rates are much higher in Myanmar than in China (*9*,*28*), persons living close to the border are at higher risk for infection. We found much a higher malaria incidence in the border area than in places farther from the border. Hot spot analysis found that high-incidence clusters of confirmed malaria were all located along the international border. Yet, the actual risk depends on the exact locations because malaria incidence rate in the border townships varied tremendously, from 0 to 1,133 cases/100,000 population annually in 2013. In addition, the hot spots of malaria changed over time and differed between *P. falciparum* and *P. vivax*. The shift of *P. falciparum* transmission hot spots might again be strongly linked to the pattern of cross-border population migration (*9*,*28*,*29*). The Getis-Ord local G_i_*(d) test has been commonly used to determine clustering of transmission hot spots of different diseases (*14*,*21*–*24*); the test can be used in even finer-scale clustering analysis, such as village- or household-level hot spot analysis (*20*,*30*,*31*). The use of spatial clustering combined with geographic information systems clearly shows the locations and the sizes of the transmission hot spots. If this method is implemented in real-time monitoring, the development of hot spots can be detected early, thus enabling targeted interventions in a timely manner. Thus, this method can become a conventional parameter in a programmatic decision-making process by disease prevention and control authorities. We emphasize that, for active case tracking at the elimination stage, such a hot spot analysis at the village or household level might be worth investigating, even though it demands more resources (*31*). In addition, the spatial shift in malaria incidence hot spots from year to year is a challenge for the national malaria control program. For the national malaria control program to identify hot spots of malaria incidence and target malaria control in a timely manner, the spatial cluster analysis will need to be performed more frequently.

Regardless of the changing epidemiology, malaria cases continued to exhibit a seasonal transmission pattern, peaking in April–July, mostly reflecting the dynamics of monthly rainfall in this region (*9*,*32*–*34*). Seasonal migration patterns also might contribute to seasonal malaria transmission, as in other parts of the Greater Mekong Subregion such as western Thailand (*35*–*38*). Therefore, measures need to be developed to strengthen surveillance of cross-border migratory human populations to prevent malaria reintroduction. One limitation of our study is that we did not differentiate indigenous from definitive imported cases because of the lack of travel history information for many persons with confirmed malaria. The clear seasonality of malaria cases and migratory populations should be considered in future malaria prediction analyses so that targeted monitoring and control efforts can be deployed more precisely.

The complex heterogeneous distribution of low but focalized and mobilized confirmed malaria transmission brings enormous challenges to malaria elimination plans. Strategies for malaria elimination in China should focus on these few transmission foci. For example, vector control measures should be enhanced in these areas; active case surveillance should be deployed to track the transmission; and more rigorous case management strategies should be implemented. In addition, Myanmar is considered the major source of parasite importation in the entire Greater Mekong Subregion (*39*,*40*), and human migration in the area plays an important role in malaria transmission in China (*35*–*38*; http://www.searo.who.int/entity/malaria/documents/Mekong_pro/en/). Therefore, future elimination efforts should focus on the effects of cross-border activities on malaria parasite transmission, and strategies should include more intensive surveillance so that prevention and control activities can be directed at hot spot regions along the China–Myanmar border.
